# CLIA-certified next-generation sequencing analysis in the cloud

**DOI:** 10.1186/1753-6561-6-S6-P54

**Published:** 2012-10-01

**Authors:** Ying Zhang, Jesse Erdmann, John Chilton, Getiria Onsongo, Matthew Bower, Kenny Beckman, Bharat Thyagarajan, Kevin Silverstein, Anne-Francoise Lamblin

**Affiliations:** 1 Research Informatics Support System, Minnesota Supercomputing Institute, University of Minnesota, Minneapolis, MN 55455, USA; 2 Division of Genetics and Metabolism, University of Minnesota, Minneapolis, MN 55455, USA; 3 Molecular Diagnostics Laboratory, University of Minnesota Medical Center-Fairview, University of Minnesota, Minneapolis, MN 55455, USA; 4 BioMedical Genomics Center, University of Minnesota, Minneapolis, MN 55455, USA; 5 Department of Laboratory Medicine and Pathology, University of Minnesota, Minneapolis, MN 55455, USA

## 

The development of next-generation sequencing (NGS) technology opens new avenues for clinical researchers to make discoveries, especially in the area of clinical diagnostics. However, combining NGS and clinical data presents two challenges: first, the accessibility to clinicians of sufficient computing power needed for the analysis of high volume of NGS data; and second, the stringent requirements of accuracy and patient information data governance in a clinical setting.

Cloud computing is a natural fit for addressing the computing power requirements, while Clinical Laboratory Improvement Amendments (CLIA) certification provides a baseline standard for meeting the demands on researchers in working with clinical data. Combining a cloud-computing environment with CLIA certification presents its own challenges due to the level of control users have over the cloud environment and CLIA's stability requirements. We have bridged this gap by creating a locked virtual machine with a pre-defined and validated set of workflows. This virtual machine is created using our Galaxy VM launcher tool to instantiate a Galaxy [http://www.usegalaxy.org] environment at Amazon with specific versions of the tools used in the workflow. The VM launcher tool can reliably recreate the same virtual machine on several cloud environments. Once a baseline virtual machine is created, the tool can launch any number of clones to analyze samples in parallel. We describe herein a pilot project as an example of a working clinical analysis pipeline. In order to validate the clinical diagnosis of diseases with a genetic cause using NGS data, patient samples were collected by Dr Bharat Thyagarajan and staff at the Molecular Diagnostics Laboratory, University of Minnesota medical center-Fairview. The patient samples were analyzed using customized hybrid-capture bait libraries to boost read coverage in low-coverage regions, followed by targeted enrichment sequencing at the BioMedical Genomics Center. The NGS data is imported to a tested Galaxy single nucleotide polymorphism (SNP) detection workflow in a locked Galaxy virtual machine on Amazon's Elastic Compute Cloud (EC2). This project illustrates our ability to carry out CLIA-certified NGS analysis in the cloud, and will provide valuable guidance in any future implementation of NGS analysis involving clinical diagnosis.

